# The Tumor Immune Microenvironment and Therapeutic Efficacy of Trastuzumab Deruxtecan in Gastric Cancer

**DOI:** 10.1158/2767-9764.CRC-24-0302

**Published:** 2025-01-14

**Authors:** Shigehiro Koganemaru, Shohei Koyama, Fumitaka Suto, Makito Koga, Koichiro Inaki, Yusuke Kuwahara, Takeo Arita, Tsuyoshi Hirata, Hiroki Goto, Naoya Wada, Maki Kobayashi, Tomoko Shibutani, Tatsuya Okabayashi, Kenji Nakamaru, Akihito Kawazoe, Yousuke Togashi, Hiroyoshi Nishikawa, Kohei Shitara

**Affiliations:** 1 Department of Experimental Therapeutics, National Cancer Center Hospital East, Kashiwa, Japan.; 2 Division of Cancer Immunology, Research Institute/Exploratory Oncology Research and Clinical Trial Center, National Cancer Center, Tokyo/Chiba, Japan.; 3 Department of Immunogenomic Medicine, Research Institute, National Cancer Center, Tokyo, Japan.; 4 Department of Respiratory Medicine and Clinical Immunology, Osaka University Graduate School of Medicine, Osaka, Japan.; 5 Translational Science Department, Precision Medicine Function, Daiichi Sankyo, Inc., Basking Ridge, New Jersey.; 6 Translational Science Department I, Precision Medicine Function, Daiichi Sankyo, Co., Ltd., Tokyo, Japan.; 7 Oncology Medical Science Department, Medical Affairs Division, Daiichi Sankyo, Co., Ltd., Tokyo, Japan.; 8 Translational Research Department, Daiichi Sankyo RD Novare Co., Ltd., Tokyo, Japan.; 9 Clinical Development Department I, Development Function, Daiichi Sankyo, Co., Ltd., Tokyo, Japan.; 10 Department of Tumor Microenvironment, Faculty of Medicine, Dentistry and Pharmaceutical Sciences, Okayama University, Okayama, Japan.; 11 Department of Immunology, Nagoya University Graduate School of Medicine, Nagoya, Japan.; 12 Department of Gastroenterology and Gastrointestinal Oncology, National Cancer Center Hospital East, Kashiwa, Japan.

## Abstract

Trastuzumab deruxtecan (T-DXd), an anti-HER2 antibody–drug conjugate with a topoisomerase I inhibitor connected by a cleavable linker, has been approved for patients with HER2-positive gastric or gastroesophageal junction tumors. This biomarker study assessed HER2 expression and immune cell infiltration in relation to the therapeutic response to T-DXd. This retrospective analysis included samples from patients treated with T-DXd in three clinical trials. We performed RNA sequencing and multiplex IHC on archival tumor samples obtained at baseline, during treatment, and after treatment. Flow cytometry was performed on tumor-infiltrating immune cells freshly isolated from tumor tissues. Samples from 28 patients were included in this study. *ERBB2* mRNA levels and CD20^+^ cell infiltration in tumors were significantly higher at baseline in responders than in nonresponders. Patients were classified into three biological groups based on their baseline tumor/stroma-infiltrating immune cell densities. Two groups reported similar response rates, but a trend was observed toward a shorter progression-free survival in the group with more immunosuppressive regulatory T cells and PD-L1 expression at baseline. T-DXd treatment tended to increase the levels of tumor-infiltrating CD8^+^ T cells and PD1^+^CD8^+^ T cells, particularly in responders. Gene expression signatures of CTL and Th cells increased during treatment, whereas signatures related to hypoxia, *MYC* targets, collagen formation, and IL-10 were downregulated. Our data suggest that HER2 expression levels and baseline tumor microenvironment characteristics correlate with T-DXd efficacy. Furthermore, this treatment may modulate tumor microenvironment immune profiles. Further validation using a larger sample size is warranted.

**Significance:**

This biomarker study explored HER2 expression levels and immune cell characteristics that may affect response to T-DXd using tumor tissue samples collected from clinical trial participants. The results suggest that HER2 expression levels and tumor characteristics before the initiation of T-DXd may correlate with the efficacy of the drug.

## Introduction

HER2 is overexpressed in approximately 21% to 32% of patients with gastric cancer or gastroesophageal junction (GEJ) cancer (1). The antibody–drug conjugate trastuzumab deruxtecan (T-DXd) is used in HER2-targeted therapy and comprises an anti-HER2 antibody, a cleavable linker, and a topoisomerase I inhibitor (2).

DESTINY-Gastric01 was a randomized, open-label, phase II trial that investigated T-DXd versus physician’s choice of chemotherapy in patients with HER2-positive (HER2^+^) gastric cancer or GEJ cancer that had progressed in response to at least two previous therapies (2). T-DXd therapy significantly improved the objective response rate (ORR) compared with physician’s choice of chemotherapy (2). Therefore, T-DXd is a novel HER2-targeted therapy approved for patients with HER2^+^ gastric cancer or GEJ cancer after trastuzumab treatment failure.

In the biomarker analysis of DESTINY-Gastric01, a correlation between baseline HER2 expression and ORR was observed (2). However, tumor responses varied even within the group of patients with HER2^+^ tumors (2, 3), which suggests that factors other than HER2-related factors might also be associated with the therapeutic effect of T-DXd.

T-cell infiltration into the tumor microenvironment (TME) has been associated with the prognosis of gastric cancer (4, 5). The infiltration of CD3^+^, CD8^+^, and CD4^+^ T cells is associated with a favorable prognosis (4), whereas that of FOXP3^+^ regulatory T cells (Tregs), an immunosuppressive subset of CD4^+^ T cells, is associated with a poor prognosis in several cancers (6). Previous studies have shown that baseline immune profiles in the TME affect chemotherapy efficacy (7). Furthermore, chemotherapy has been shown to modulate the TME (8). Unfavorable immune features of the TME are more pronounced in patients with HER2^+^ tumors (9–11). In preclinical models, T-DXd treatment has been shown to increase tumor-infiltrating T cells and activate dendritic cells (DC). Additionally, a synergistic antitumor efficacy has been observed when T-DXd treatment was combined with an anti–PD-1 antibody (12).

However, the relationship between T-DXd efficacy and baseline tumor immune profiles and the activity of T-DXd in modulating TME immune profiles have not been investigated in clinical samples. This biomarker study aimed to explore HER2 expression and immune cell infiltration in relation to the clinical efficacy of T-DXd in patients with gastric cancer and verify whether T-DXd exhibits immunomodulatory activity.

## Materials and Methods

### Study design

This was a retrospective analysis of tumor or blood biomarkers from patients who had been treated with T-DXd during phase I, phase II clinical trials, and the expanded access clinical trials (2, 13–15). The eligibility criteria for each trial have been described previously (2, 13–15). Briefly, patients were enrolled if they had HER2^+^ or HER2-low status (IHC score 2+/ISH-negative), as revealed by local (NCT02564900 and jRCT2080225138) or central (NCT03329690) assessments of archival tumor tissues. Patients treated with T-DXd (1.6 mg/kg) in the phase I trial (13) were excluded from the analysis because this dose was extremely low compared with the approved dose. Patients with HER2-low status (IHC score 2+/ISH-negative) and no prior trastuzumab therapy were also excluded from the analysis.

All patients provided written informed consent before T-DXd treatment. Furthermore, patients who underwent biomarker analysis provided written informed consent for the analysis (Institutional Review Board approval number: 2018-231). The study protocol was approved by the Institutional Review Board at the National Cancer Center Japan and conducted in accordance with the ethical principles outlined in the Declaration of Helsinki.

### Sample collection

Exploratory biomarker data were collected from the tumor and blood samples of patients who participated in the NCT02564900, jRCT2080225138, and NCT03329690 trials of T-DXd at the National Cancer Center Hospital East, Japan (2, 13–15). In the present study, all baseline tumor tissues were analyzed to evaluate the correlation of HER2 expression and immune cell densities with T-DXd efficacy. Additionally, fresh tumor samples were collected immediately before, during treatment (at the time of cycle 3), and at the end of T-DXd treatment, if feasible. Tumor tissues for the baseline analysis were collected from eligible patients after they initiated trastuzumab treatment. Among the predefined parameters, those with data of ≥6 patients were subjected to further analyses. Samples for the end of T-DXd treatment were not subjected to further analyses because some of these specimens were not collected immediately after the treatment completion, leading to a diversity in sample collection timing. A flowchart of the sample collection process is presented in Supplementary Fig. S1.

RNA sequencing (RNA-seq) biomarker analysis was performed on tumor biopsies at baseline, during treatment, and at the end of treatment to determine *ERBB2* expression levels, comprehensive gene expression profiles, and immune-related gene profiles. Samples obtained at the end of T-DXd treatment were excluded from further RNA-seq analysis because of a diversity in the timing of sample collection.

Total RNA was extracted from formalin-fixed, paraffin-embedded tumor tissue block slices using the AllPrep DNA/RNA FFPE kit (QIAGEN; Cat. No. 80234) and QIAcube (QIAGEN). The extracted RNA was quantified and evaluated for purity using the 4200 TapeStation system (Agilent Technologies, Inc.) and a Qubit fluorometer (Thermo Fisher Scientific).

A cDNA library was prepared from the extracted tumor RNA for directional sequencing, including the depletion of reverse-transcribed ribosomal RNA and unique dual indices, using the SMARTer Stranded Total RNA-Seq Kit v.2—Pico Input Mammalian (Takara Bio Inc.; Cat. No. Z4412N). The quality and quantity of the prepared cDNA library were evaluated using the 4200 TapeStation system, according to the manufacturer’s protocol. The prepared libraries were pooled and sequenced on a NextSeq 500 or NextSeq 550 next-generation sequencer (Illumina Inc.) with 75 bp from single ends, and the target number of reads per sample was 10 million. Using STAR software (version 2.5.3a, RRID: SCR_004463), the sequenced reads were aligned to the human genome reference (hg38). Transcripts per million, the number of mapped reads in each gene, and fragments per kb of exon per million mapped reads in each gene were estimated using RSEM software (version 1.3.0, RRID: SCR_000262).

Differential expression analysis was performed in R (version 3.5.1, RRID: SCR_001905) using the “limma” package (version 1.38.3, RRID: SCR_010943; ref. 16). Gene set enrichment analysis (GSEA) was performed in R using the “fgsea” package (version 1.24.0, RRID: SCR_020938; bioRxiv 060012). The gene sets used for the C2 canonical pathway and hallmark enrichment analysis were downloaded from the Molecular Signatures Database (http://www.gsea-msigdb.org, RRID: SCR_016863).

### IHC

HER2 IHC was retrospectively performed to confirm the HER2 expression status in this study. For HER2 detection, the tissue sections were stained with an anti-HER2/neu (4B5) rabbit monoclonal antibody (clone: 4B5; VENTANA pathway HER2, RRID: AB_2921204) as the primary antibody. The percentage of tumor cells with HER2 staining and staining intensity were evaluated to determine the HER2 score. Based on the American Society of Clinical Oncology/College of American Pathologists guidelines (17), HER2 status was classified into four groups: IHC 3+ (intensity 3 staining observed in >10% of tumor cells), IHC 2+ (intensity 2 staining in >10% of tumor cells), IHC 1+ (intensity 1 staining in ≤10% of tumor cells), and IHC 0 (no staining was observed).

For the detection of DC markers, tissue sections were stained with an anti-CD1a mouse monoclonal antibody (clone: O10; Invitrogen, RRID: AB_10943672) and an anti-CD208 rabbit polyclonal antibody (Invitrogen, RRID: AB_2791221) using the Leica BOND RX automated slide stainer (Leica Biosystems). The human tonsil was used as the positive control, and mouse immunoglobulin (IgG) isotype control (equivalent concentration) was used as the negative control. The positive cell count per unit area (mm^2^) of the entire tissue area was manually evaluated by a trained pathologist. Hematoxylin and eosin–stained slides were used as references for histologic analysis.

Tissue sections were also subjected to multiplex IHC (mIHC) using the Opal 7-Color Automation IHC kit (Akoya Biosciences; Cat. No. OP-000003) and primary antibodies against PD-1 (clone: NAT105; Abcam, RRID: AB_3492087), PD-L1 (clone: SP142; Abcam, RRID: AB_2827816), CD4 (clone: 4B12; Leica Biosystems, RRID: AB_10554438), CD8 (clone: C8/144B; Dako, RRID: AB_2075537), CD20 (clone: L26; Dako, RRID: AB_3075456), CD68 (clone: 514H12; Dako), CD163 (clone: 10D6; Leica Biosystems, RRID: AB_2756375), FOXP3 (clone: D608R; Cell Signaling Technology, RRID: AB_2797979), and pan-cytokeratin (clone: AE1/AE3; Dako). CD4, CD8, CD20, PD-1, PD-L1, and FOXP3 were used as markers to determine the phenotype of tumor-infiltrating T and B cells, and CD68, CD163, and PD-L1 were used as markers to determine the phenotype of tumor-associated macrophages (TAM). Pan-cytokeratin was used as a tumor cell marker. The human tonsil was used as the positive control. Briefly, all tissue sections were stained using the autostainer BOND RX (Leica Biosystems), according to the manufacturer’s instructions, and subjected to mIHC using the Opal 7-Color Automation IHC kit and BOND Research Detection kit (Leica Biosystems, Cat. No. DS9455). The stained slides were mounted using the ProLong Diamond Antifade Mountant (Thermo Fisher Scientific; Cat. No. P36961).

### Image analysis

The stained mIHC slides were subjected to seven-color multispectral image analysis using the Automated Quantitative Pathology Imaging System, Vectra Polaris (version 1.0.10; PerkinElmer). The whole slide was scanned at 20× magnification. The top five tumor-infiltrating lymphocyte (TIL) and TAM hotspot fields were selected as regions of interest (resolution of 0.5 μm per pixel, 931 μm × 698 μm) using Phenochart viewer (version 1.0.12; PerkinElmer, RRID: SCR_019156). Image files generated by Vectra Polaris were analyzed using the image analysis software inForm (version 2.4.11; PerkinElmer, RRID: SCR_019155). Seven-color image preparation, trainable tissue segmentation, adaptive cell segmentation, and “score” or “phenotyping” methods were performed using inForm software. Batch analysis was performed on all high-power images using an algorithm designed for representative lesions.

Log10 transformation was applied to the cell densities before plotting a heatmap and clustering analysis. A constant (0.1) value was added to allow log10 transformation of zero. A heatmap was generated using the “ComplexHeatmap” package (version 2.3.1, RRID: SCR_017270; refs. 18, 19).

### Flow cytometry

Flow cytometry (FCM) analysis of TILs was conducted to evaluate intratumoral immune cells at baseline, during treatment, and at the end of treatment. Sample preparation for FCM, staining, and analysis was performed as previously described (20, 21). The antibodies used in this study are summarized in Supplementary Table S1. Briefly, the cells were washed with PBS containing 2% FCS and stained with surface antibodies and a fixable viability dye (BD Biosciences; Cat. No. 565388). Subsequently, intracellular staining was performed using the FOXP3/Transcription Factor Staining Buffer set (Thermo Fisher Scientific; Cat. No. 00-5523-00), according to the manufacturer’s instructions. After washing, the cells were analyzed with the LSRFortessa cell analyzer (BD Biosciences) and FlowJo software (BD Biosciences, RRID: SCR_008520).

### Outcomes

The ORR, best overall response (BOR), overall survival (OS), and progression-free survival (PFS) of patients enrolled in each study were evaluated. Tumor response was assessed in patients with measurable lesions according to the RECIST guidelines (version 1.1). Responders were defined as patients who achieved a complete response or partial response, and nonresponders were defined as patients who achieved stable disease or had progressive disease. BOR was defined as the best response from the start of treatment until disease progression. OS was defined as the time from the first treatment cycle to death from any cause. PFS was defined as the time from treatment initiation to disease progression or death from any cause.

### Statistical analysis

Statistical analysis was performed using R (version 3.5.1). The Wilcoxon rank-sum test was performed to compare the data of responders and nonresponders for each biomarker. The Spearman rank correlation test was performed to evaluate the relationships between biomarkers. Point estimates and two-sided 95% exact binomial confidence intervals (CI) were calculated for ORR in each subgroup. The Kaplan–Meier method was used to estimate median event times, with two-sided 95% CIs calculated using the Brookmeyer–Crowley method. The median count of each cell type was used as the cutoff value to establish high and low subgroups for each parameter. *P* < 0.05 was considered statistically significant. This was a hypothesis-free, exploratory, biomarker study; therefore, the sample size required for statistical detection was not determined, and a retrospective exploratory study was conducted using all samples available for analysis.

### Data availability

The data generated in this study are publicly available in Gene Expression Omnibus at GSE262648.

## Results

### Patient characteristics

We enrolled 28 patients for this study. The median age was 65.5 years (range: 37–80), and 78.6% of the patients were men. Of the 28 patients, 20 were responders to the treatment (complete response or partial response) and 8 were nonresponders (stable disease or progressive disease), as per RECIST version 1.1.

Seventeen patients did not receive previous immunotherapy, seven had previously received anti–PD-1/PD-L1 therapy, and four had an unknown history of anti–PD-1/PD-L1 therapy because they had participated in a placebo-controlled trial. A summary of the patient characteristics is presented in Supplementary Table S2.

### Relationship between baseline HER2 expression and therapeutic response to T-DXd

**Figure 1:**
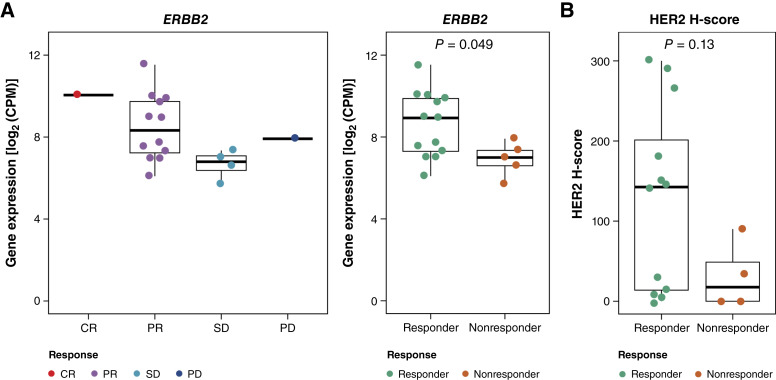
Associations between pretreatment HER2 expression levels and therapy response to T-DXd. **A, ***ERBB2* mRNA expression levels, as determined using RNA-seq. **B,** HER2 H-score, as determined using IHC. CPM, counts per million; CR, complete response; PD, progressive disease; PR, partial response; SD, stable disease.

We analyzed the baseline *ERBB2* expression using RNA-seq and HER2 using IHC to determine the relationship between HER2 expression and therapeutic response to T-DXd. In 18 patients whose tumor specimens were collected after trastuzumab treatment, the tumor tissues were analyzed as baseline to accurately assess the correlation between HER2 expression and T-DXd efficacy. The response rates were similar in these 18 patients: 13 responders and five nonresponders. We observed a positive association between HER2 status and BOR (Fig. 1A). A significantly higher *ERBB2* mRNA expression level was observed in responders than in nonresponders (*P* = 0.049). Furthermore, the HER2 H-score was numerically higher in responders than in nonresponders, as assessed by HER2 IHC (*P* = 0.13; Fig. 1B).

### Relationship between baseline immune cell densities and BOR

**Figure 2:**
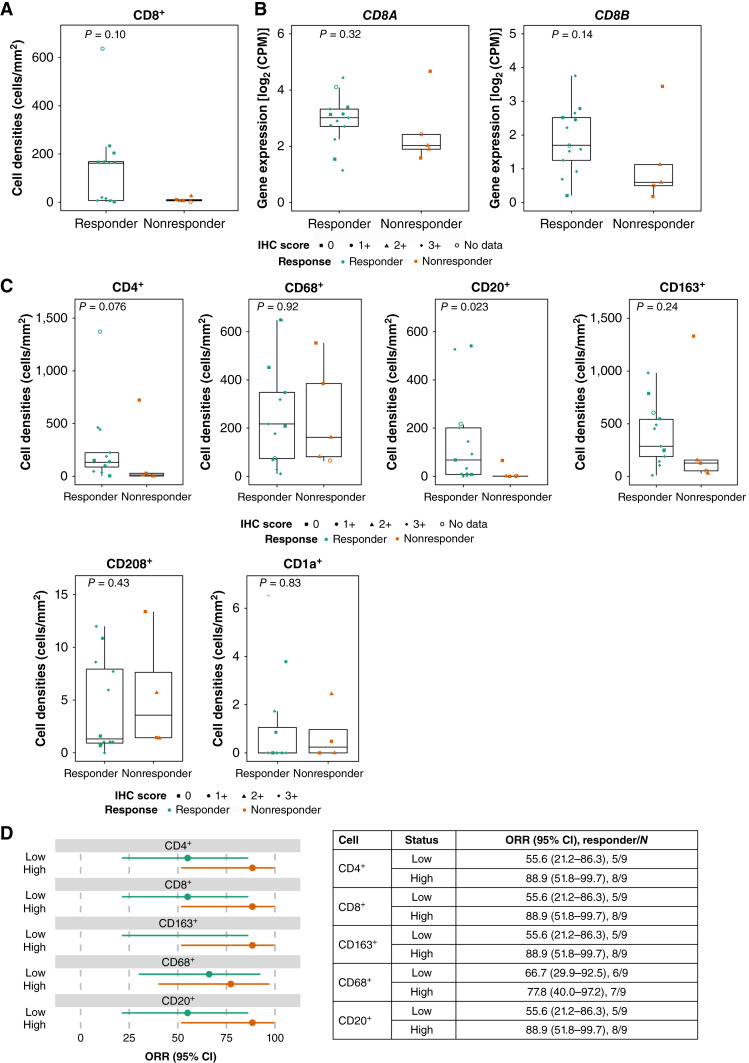
Immune infiltration in tumors at baseline and association with response to T-DXd therapy. **A,** Tumoral CD8^+^ cell densities, as determined using mIHC, at baseline in responders and nonresponders. **B, ***CD8A* and *CD8B* mRNA expression levels, as determined using RNA-seq, in responders and nonresponders. CPM, counts per million. **C,** Relationship between immune cell densities for cells expressing CD4, CD68, CD20, CD163, CD208, and CD1a, as determined using mIHC and IHC. **D,** Relationship between immune cell densities and T-DXd efficacy. Patients with high vs. low immune cell densities were grouped based on the median value.

Figure 2A and B show the relationship between baseline CD8^+^ T-cell density, determined using mIHC, and T-DXd efficacy, as well as between *CD8A/B* expressions, determined using RNA-seq, and T-DXd efficacy. A numerically higher infiltrating CD8^+^ cell density was observed in the TME of responders (median: 161.9 cells/mm^2^) than in that of nonresponders (median: 7.1 cells/mm^2^; *P* = 0.10). This trend was consistent when the tumor tissues were stratified by *CD8A* or *CD8B* expression.

A significant association was observed between T-DXd efficacy and the median number of baseline tumor-infiltrating CD20^+^ cells (67.8 vs. 0.9 cells/mm^2^) in responders versus nonresponders (*P* = 0.023). No significant associations were observed between T-DXd efficacy and the median number of baseline tumor-infiltrating CD4^+^ (132.7 vs. 12.2 cells/mm^2^; *P* = 0.076), CD68^+^ (217.3 vs. 161.9 cells/mm^2^; *P* = 0.92), CD163^+^ (286.8 vs. 127.7 cells/mm^2^; *P* = 0.24), CD208^+^ (1.3 vs. 3.6 cells/mm^2^; *P* = 0.43), or CD1a^+^ (0 vs. 0.2 cells/mm^2^; *P* = 0.83) immune cells in responders versus nonresponders (Fig. 2C).

No clear association was observed between the HER2 H-score and CD8^+^ cell densities (*r* = −0.056, *P* = 0.84; Supplementary Fig. S2A) or between the *ERBB2* mRNA expression level and *CD8A* expression (*r* = 0.42, *P* = 0.086; Supplementary Fig. S2B). When the patients were stratified by prior immunotherapy status, there were no notable differences in the results between the subgroups (Supplementary Fig. S3A–S3C).

Figure 2D shows the relationship between immune cell densities (CD4, CD8, CD163, CD68, and CD20) and T-DXd efficacy (ORR; patients with high vs. low immune cell densities were grouped based on the median value). A numerically higher ORR was observed in patients with high baseline tumor-infiltrating immune cell densities than in those with low immune cell densities, with a small difference for CD68^+^ cells. ORR was higher for high versus low CD4^+^ (88.9% vs. 55.6%), CD8^+^ (88.9% vs. 55.6%), CD163^+^ (88.9% vs. 55.6%), and CD20^+^ (88.9% vs. 55.6%) cell densities (ORR in the intention-to-treat population: 72.2%).

**Figure 3:**
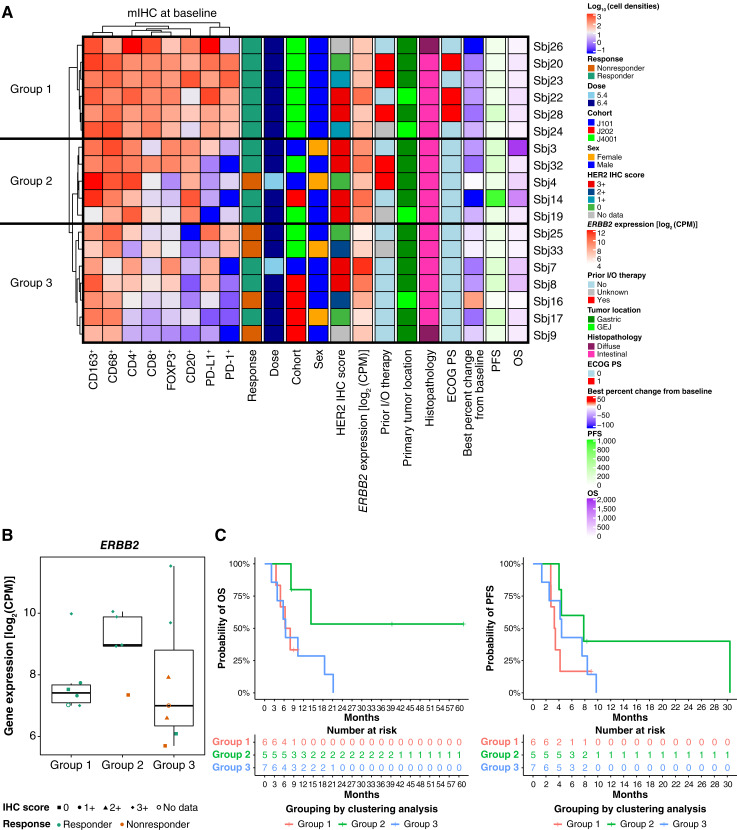
Unsupervised hierarchical clustering analysis based on cell densities of evaluated immune cells at baseline. **A,** Heatmap depicting unsupervised hierarchical clustering results and characteristics of the three observed groups. **B, ***ERBB2* expression levels in the three groups obtained from clustering. **C,** Kaplan–Meier OS and PFS curves of groups 1, 2, and 3 grouped by unsupervised hierarchical clustering based on cell densities of the evaluated immune cells. CPM, counts per million; ECOG PS, Eastern Cooperative Oncology Group performance status; I/O, immunotherapy.

Based on baseline tumor/stroma-infiltrating immune cell densities, we classified the patient population into three groups by unsupervised hierarchical clustering, which revealed a higher ORR in groups 1 (*n* = 6) and 2 (*n* = 5), in which the overall immune cell densities were higher than those in group 3 (*n* = 7; Fig. 3A). Group 1 included three patients with prior immunotherapy, two patients without prior immunotherapy, and one patient with unknown prior immunotherapy status, whereas group 2 included two patients each with and without prior immunotherapy and one patient with unknown prior immunotherapy status (Fig. 3A). A higher ORR was observed in groups 1 (100.0%) and 2 (80.0%), which had higher levels of CD4^+^, CD8^+^, and CD20^+^ cells than group 3 (42.9%). *ERBB2* expression was numerically higher in group 2 than in groups 1 and 3 (Fig. 3B). The longest OS and PFS were observed in group 2 [median OS (95% CI): not reached (NR; 8.12–NR); median PFS (95% CI): 7.82 months (4.01–NR)], which had higher levels of CD4^+^, CD8^+^, or CD20^+^ cells than group 3 [median OS (95% CI): 6.44 months (2.10–18.5); median PFS (95% CI): 4.41 months (1.35–8.38); Supplementary Fig. S4] and lower levels of PD-1^+^, FOXP3^+^, PD-L1^+^ cells, and PD-1^+^FOXP3^+^CD4^+^ cells than group 1 [median OS (95% CI): 7.10 months (3.52–NR); median PFS (95% CI): 3.37 months (2.73–NR); Fig. 3A and C; Supplementary Fig. S4]. The proportion of patients with prior immunotherapy was higher in group 1 than in group 2 (Fig. 3A), but the median PFS was longer in group 2 than in group 1 (Fig. 3C), which indicates that prior immunotherapy status may not affect interpretation of the results. GSEA results revealed that the gene signatures related to allograft rejection, IFN-γ response, Th1–Th2 pathway, and CTL pathway were higher, whereas those related to fatty acid metabolism, oxidative phosphorylation, and cholesterol biosynthesis were lower in group 2 than in group 1 at baseline (Supplementary Fig. S5).

### Changes in immune cell densities after T-DXd treatment

**Figure 4:**
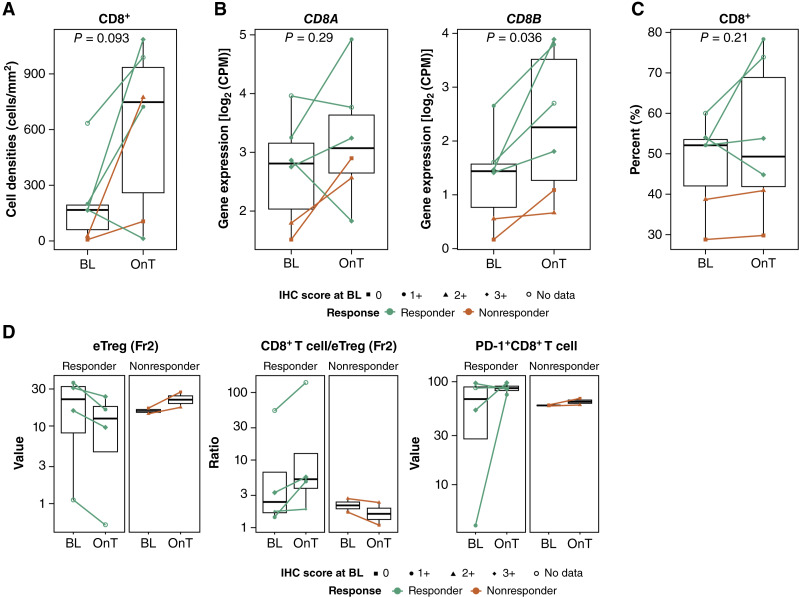
Tumor-infiltrating immune cell densities at baseline and in response to T-DXd therapy in responders and nonresponders. **A,** Tumor-infiltrating CD8^+^ T-cell densities, as determined using mIHC. **B,** Tumoral *CD8A* and *CD8B* expression, as determined using RNA-seq. **C,** Percentage of CD8^+^ cells in CD3^+^ at baseline, as determined using FCM. **D,** Densities of eTregs (Fr2) and PD-1^+^CD8^+^ T cells and the CD8^+^ T-cell/eTreg ratio (Fr2). BL, baseline; CPM, copies per million; eTreg, effector Treg; OnT, on treatment.

A trend was observed toward an increase in CD8^+^ cell density, determined using mIHC (*P* = 0.093); *CD8A/B* expression, determined using RNA-seq (*P* = 0.29 and *P* = 0.036, respectively); and the frequency of CD8^+^ cells within the CD3^+^ population among TILs (*P* = 0.21) during T-DXd treatment (Fig. 4A–C). Furthermore, CD8^+^, CD4^+^, and CD163^+^ cell densities showed a trend of higher levels during T-DXd treatment (Fig. 4A; Supplementary Fig. S6).

Although this study had a small sample size, the relative TIL ratio of CD8^+^ T cells to FOXP3^high^CD45RA^−^CD4^+^ effector Tregs was numerically increased during T-DXd treatment in responders but not in nonresponders, as revealed using FCM. Furthermore, there was a trend toward increased levels of PD1^+^CD8^+^ T cells during T-DXd treatment in responders compared with nonresponders (Fig. 4D).

### Gene signature analysis during T-DXd treatment

We performed gene expression analysis of the tumor samples collected during treatment. Hallmark GSEA revealed a decrease in *MYC* targets and hypoxia signature scores during T-DXd treatment (Supplementary Fig. S7A).

The C2 canonical pathway in GSEA showed that the CTL pathway signature and Th 1/2 cell pathway were highly expressed, whereas the collagen formation and IL-10 signaling pathways were expressed at lower levels in samples collected during treatment than in baseline samples (Supplementary Fig. S7B).

## Discussion

In this exploratory biomarker analysis of samples obtained from clinical trials evaluating T-DXd efficacy in patients with gastric cancer (2, 13–15), we assessed HER2 biomarker status and its association with clinical outcomes. A previous study has reported an association between HER2 expression and the efficacy of HER2-targeted therapy, consistent with the findings of this study (22). A reduction in HER2 expression from HER2^+^ to HER2-negative has been reported in approximately 29% of patients with gastric cancer receiving trastuzumab treatment (23, 24). Therefore, tumor tissues collected after trastuzumab treatment were used in this study to accurately evaluate the correlation between HER2 expression and T-DXd efficacy. Although most samples were HER2^+^ at the time of clinical study enrollment, several patients with HER2-negative (IHC 1+ and IHC 0) gastric cancer were examined in this study.

Tumor-infiltrating CD8^+^ T cells and CD68^+^ TAMs in resected gastric cancer are predictive of the postoperative prognosis and benefits of adjuvant chemotherapy (7), whereas FOXP3^+^ T-cell infiltration is associated with worse outcomes (25). In addition, an insufficient immune microenvironment in gastric cancer is associated with HER2-positivity or tumors having *ERBB2* genetic aberrations (9–11). In this study, we found a nonsignificant trend between various baseline immune cell densities and T-DXd efficacy and detected a significant difference between baseline CD20^+^ cell densities in responders and nonresponders. Previous reports have shown that B-cell infiltration, in particular, memory B cell and plasma cell infiltration, is associated with a superior prognosis in gastric cancer (26, 27), whereas there are also reports of patients with high peritoneal TIGIT^+^CD20^+^ B-cell infiltration having inferior clinical outcomes (28). Additionally, tertiary lymphoid structures (TLS) have been associated with response to anti–PD-1 immunotherapy (29) in cancer, although in gastric cancer, it has been shown that although mature TLS correlate with better outcomes, immature TLS correlate with poor outcomes (30). In this study, we evaluated immune cells using biopsy samples, and therefore, it was challenging to conduct a detailed analysis of TLS or TLS-like lymphoid tissues. Furthermore, T-DXd responders may have a more favorable modulation of the TME induced by a prior therapeutic regimen. Further evaluation of B-cell immune responses in biopsy samples is warranted.

In this study, we used unsupervised hierarchical clustering to classify the patients based on their baseline immune cell characteristics. This analysis revealed that group 3, which had low T- and B-cell densities, had a numerically lower ORR than groups 1 and 2, which had higher lymphocyte densities. This suggests that the baseline immune cell profiles may be involved in the mechanism of T-DXd efficacy. Although the ORR was comparable between groups 1 and 2, the median PFS was longer in group 2 than in group 1. The density of PD-1^+^FOXP3^+^CD4^+^ cells and PD-L1–expressing cells was higher in group 1 than in group 2, suggesting that immunosuppressive Tregs and PD-L1 expression by the tumor may impede long-term responses to T-DXd. Furthermore, the fatty acid metabolism signature was enriched in group 1 compared with group 2. We previously reported that fatty acid production by cancer cells is sufficient for Treg survival and immunosuppressive function (31–34), which may explain the higher number of Tregs observed in group 1 than in group 2.

The CD8^+^ T-cell/Treg ratio is a beneficial prognostic factor in chemotherapy and immunotherapy (35, 36). In this study, we found that T-DXd treatment resulted in a trend toward increased levels of tumor-infiltrating CD8^+^ T cells and other immune cells. In responders, the CD8^+^ T-cell/Treg relative ratio tended to increase during T-DXd treatment, an effect that was not observed in nonresponders. Chemotherapy can induce immunogenic cell death, which potentially promotes tumor-specific antigen presentation and T-cell accumulation, and is associated with the optimization of the tumor immune microenvironment after chemotherapy (37). Furthermore, a preclinical study in a human HER2-expressing murine colorectal cancer model reported an increase in the levels of tumor-infiltrating DCs and CD86^+^ and CD8^+^ T cells induced by T-DXd treatment, suggesting that this treatment may enhance antitumor immunity and induce immunogenic cell death (12).

T-DXd treatment decreased PD-L1 expression on the tumor cells of patients with breast cancer in the phase II DAISY study (38). In gastric cancer, previous biomarker research from the phase II DESTINY-Gastric01 study has correlated HER2 expression with therapeutic response to T-DXd (39). However, limited data on the immune response to T-DXd have previously been reported. In our study, higher levels of PD1^+^CD8^+^ T cells were detected during T-DXd treatment, which have been reported to be associated with a repertoire of clonally expanded tumor-reactive cells and the favorable efficacy of PD-1 inhibitors (36, 40, 41). This trend was particularly pronounced in responders, suggesting that T-DXd enhanced the immunostimulatory activity. These results demonstrate that T-DXd enhances immune activity and provide a rationale for combination therapy of T-DXd and ICIs in clinical trials. Results of several ongoing clinical trials evaluating T-DXd in combination with ICIs are awaited (42, 43).

Our gene expression analysis using RNA-seq showed that CTL and Th cell signatures were higher during T-DXd treatment than at baseline, indicating that T-DXd might activate T-cell immunity. This result is consistent with our finding of an increase in immune cell densities, observed using mIHC. Moreover, hypoxia, *MYC* targets, collagen formation, and IL-10 signatures were reduced during T-DXd treatment compared with baseline, suggesting that T-DXd might switch the immunosuppressive TME to an immunogenic TME (44–46). In this study, a decrease in effector Tregs was observed in responders during T-DXd treatment, whereas nonresponders showed an opposite trend. This suggests that, apart from HER2 expression, immunomodulation may contribute to the persistence of the therapeutic effects of T-DXd in gastric cancer, as baseline analysis showed the lack of correlation between HER2 expression and any immune parameters.

This is the first study to assess the tumor immune microenvironment in patients with gastric cancer treated with T-DXd. The limitations of this study are its small sample size, the collection of tumor tissue and blood samples from three different clinical studies, and its single-center and retrospective design. In addition, specimens for the end of T-DXd treatment were not analyzed further because of a diversity in sample collection time settings, which may affect data interpretation. Furthermore, it is considered that factors such as HER2 expression, patients’ history of prior immunotherapies, and patient background (Eastern Cooperative Oncology Group performance status) may have influenced T-DXd efficacy. However, due to the limited sample size, analysis of covariance including these factors could not be conducted. Further validation of these results using a larger sample size is necessary. Finally, as all patients in this study were treated with T-DXd, potential effects of chemotherapy drugs on the immune response could not be evaluated.

In summary, our findings suggest that HER2 expression levels are correlated with the antitumor activity of T-DXd. Additionally, our data suggest that the baseline tumor immune environment may be associated with T-DXd efficacy, with T-DXd treatment tending to increase tumor-infiltrating CD8^+^ cell densities in patients with gastric cancer. A validation study in a large population is warranted to confirm our data and further clarify the immunomodulatory mechanisms induced by this therapy, which will provide insights into potential combination strategies with immunotherapies aimed at improving patient outcomes.

## Supplementary Material

Supplementary Figures and Tables Supplementary Figures and Tables.
