# Supplementary material for: The Tumor Immune Microenvironment and Therapeutic Efficacy of Trastuzumab Deruxtecan in Gastric Cancer
Source: Cancer Res Commun. 2025 Jan 14;5(1):84–93. doi: 10.1158/2767-9764.CRC-24-0302 (PMC11729160; doi:10.1158/2767-9764.CRC-24-0302)
Supplement: Supplementary Figures and Tables [file crc-24-0302_supplementary_figures_and_tables.docx]

**Supplementary Files**

**Supplementary Tables:**

| **Supplementary Table S1. Antibodies used for flow cytometry experiments** | | | |
| --- | --- | --- | --- |
| **Molecule** | **Clone** | **Supplier** | **Conjugation** |
| Human PD-1 | MIH4 | BD Biosciences | BV421 |
| Human CD3 | UCHT1 | BD Biosciences | BV650 |
| Human CD45RA | HI100 | BioLegend | BV711 |
| Human CD8a | RPA-T8 | BioLegend | BV785 |
| Human CD4 | SK3 | BD Bioscience | BUV496 |
| Human CD45 | HI30 | BD Biosciences | BUV805 |
| Human FOXP3 | 236A/E7 | Thermo Fisher Scientific | PE |
| Viability dye |  | BD Biosciences | APC-H7 |

*PD-1, programmed cell death protein 1.*

| **Supplementary Table S2: Patient characteristics** | |
| --- | --- |
| **All patients (N=28)** | |
| **Characteristics** | **n (%)** (unless specified otherwise) |
| Median age, years [range] | 65.5 [37-80] |
| Sex |  |
| Male | 22 (78.6) |
| Female | 6 (21.4) |
| ECOG PS |  |
| 0 | 25 (89.3) |
| 1 | 3 (10.7) |
| HER2 status (at clinical study enrollment) |  |
| IHC 3+ | 23 (82.1) |
| IHC 2+ | 5 (17.9) |
| HER2 status (at baseline in the present study) |  |
| IHC 3+ | 8 (50.0) |
| IHC 2+ | 2 (12.5) |
| IHC 1+ | 2 (12.5) |
| IHC 0 | 4 (25.0) |
| Clinical trial ID |  |
| NCT02564900 | 6 (21.4) |
| NCT03329690 | 7 (25.0) |
| JRCT2080225138 | 15 (53.1) |
| T-DXd dose |  |
| 5.4 mg/kg | 2 (7.1) |
| 6.4 mg/kg | 26 (92.9) |
| Prior anti-HER2 therapy |  |
| Yes | 28 (100.0) |
| No | 0 (0.3) |
| Prior anti–PD-1/PD-L1 therapy |  |
| Yes | 7 (25.0) |
| No | 17 (60.7) |
| Unknown | 4 (14.3) |

*ECOG PS, Eastern Cooperative Oncology Group performance status; IHC, immunohistochemistry; PD-1, programmed cell death protein 1; PD-L1, programmed death-ligand 1; T-DXd, trastuzumab deruxtecan.*

**Supplementary Figures:**


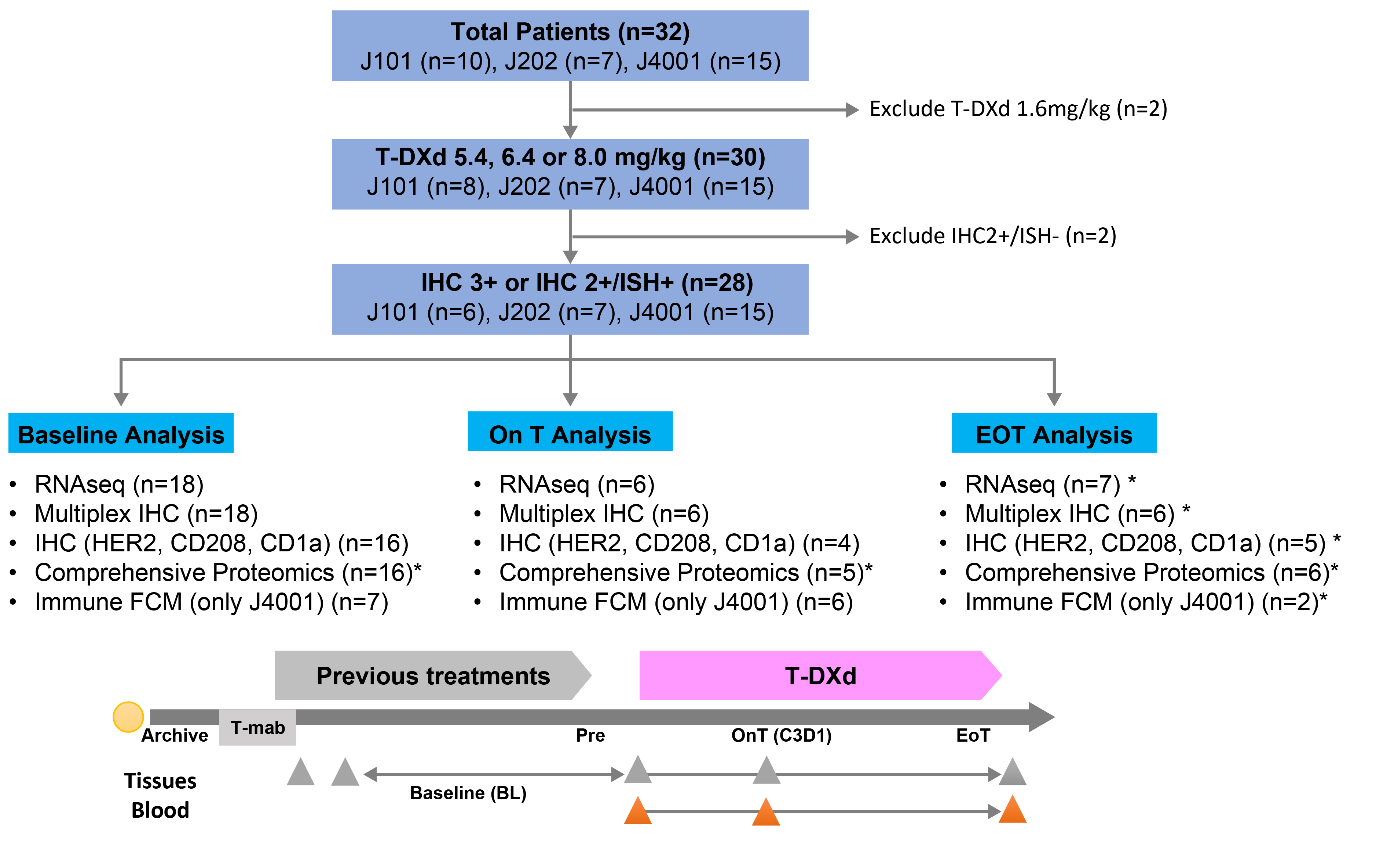

**Supplementary Figure S1. Flow chart of the sample collection process.** Tumor tissues for the baseline analysis were collected from eligible patients after they initiated trastuzumab treatment. *Although data were obtained, further analyses were not performed. *BL, baseline; C3D1, Cycle 3, Day 1; EoT, end of treatment; FCM, flow cytometry; IHC, immunohistochemistry; ISH, in situ hybridization; J101, study code for NCT02564900; J202, study code for NCT03329690, J4001, study code for jRCT2080225138; OnT, on treatment; RNA-seq, RNA sequencing; T-DXd, trastuzumab deruxtecan; T-mab, trastuzumab.*

**
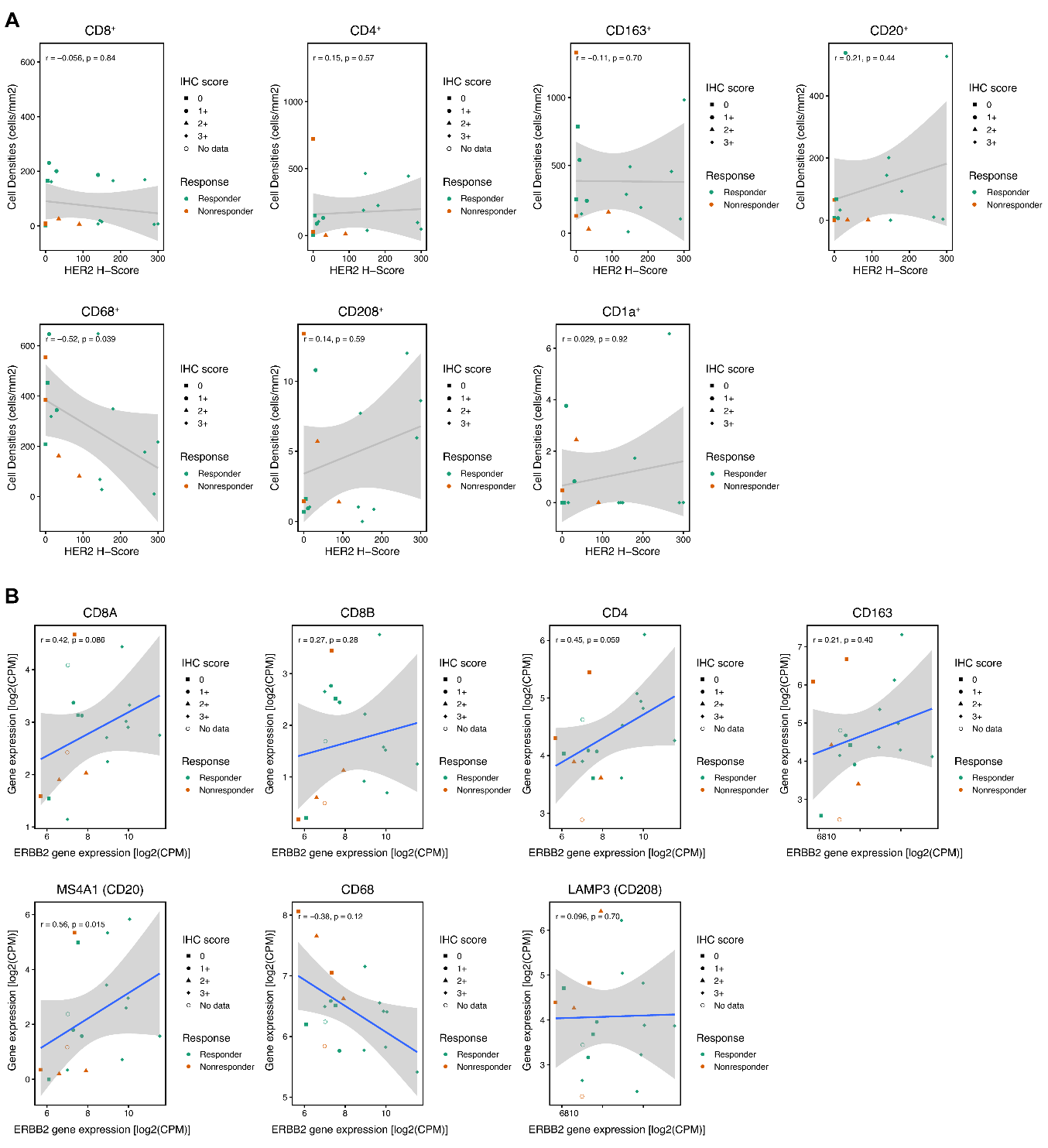
**

**Supplementary Figure S2. Associations between HER2 expression and immune cell infiltration. A,** Association between HER2 expression and immune cell markers, as determined using IHC. **B,** Association between HER2 expression and immune cell markers, as determined using RNA-seq. *CPM, counts per million; IHC, immunohistochemistry; RNA-seq, RNA sequencing.*


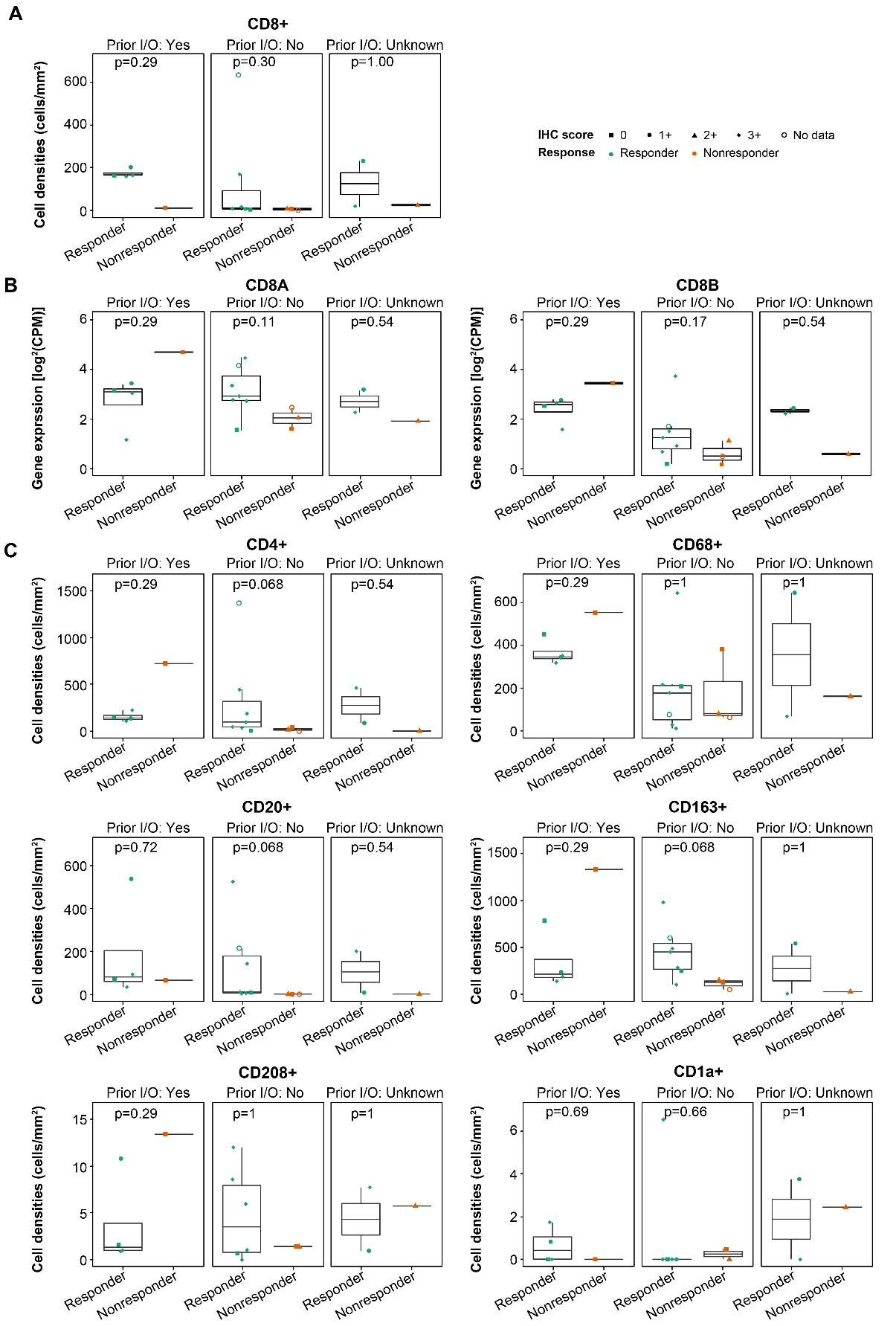


**Supplementary Figure S3. Immune infiltration in tumors at baseline and association with response to T-DXd therapy stratified by prior immunotherapy status. A,** Tumoral CD8^+^ cell densities, as determined using mIHC, at baseline in responders and nonresponders. **B,** *CD8A* and *CD8B* mRNA expression levels, as determined using RNA-seq, in responders and nonresponders. **C,** Relationship between immune cell densities for cells expressing CD4, CD68, CD20, CD163, CD208, and CD1a, as determined using mIHC and IHC. *CPM, counts per million; IHC, immunohistochemistry; I/O, immunotherapy; mIHC,* *multiplex IHC; RNA-seq, RNA sequencing; T-DXd, trastuzumab deruxtecan.*


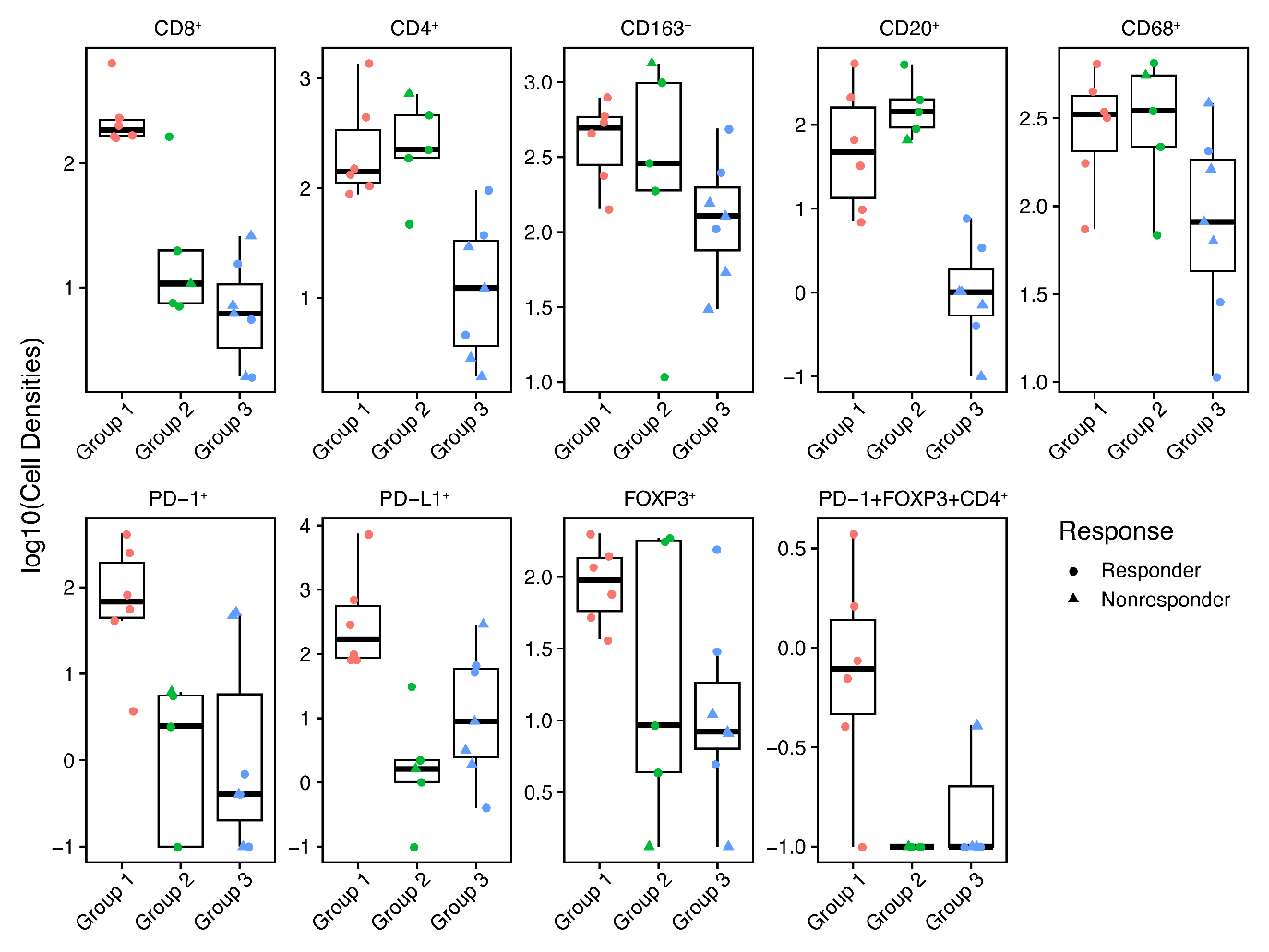


**Supplementary Figure S4. Immune cell densities in the three groups obtained from clustering analysis.** *PD-1, programmed cell death protein 1; PD-L1, programmed death-ligand 1.*

*
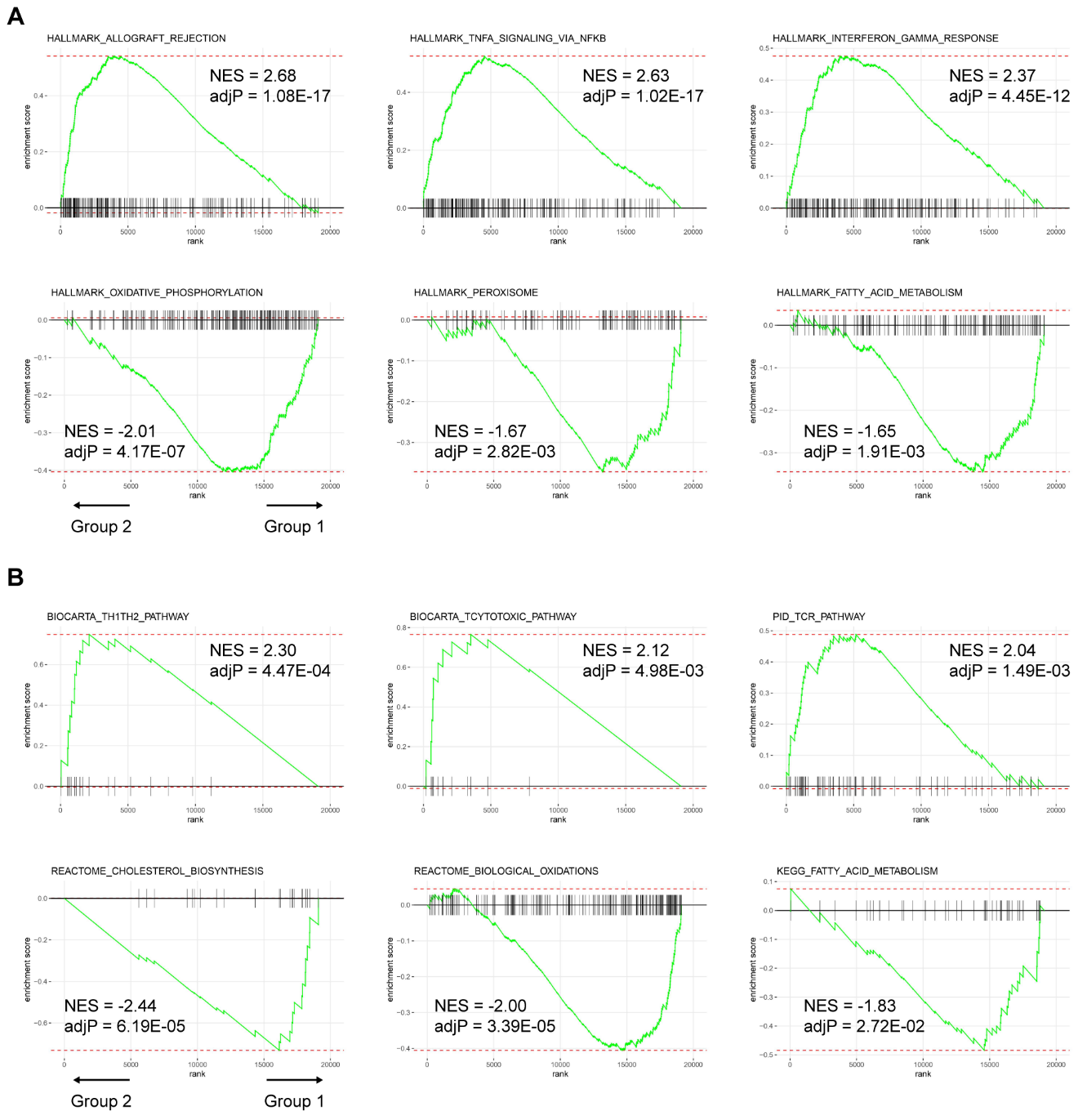
*

**Supplementary Figure S5. GSEA between Group 2 and Group 1 at baseline (unsupervised hierarchical clustering based on cell densities of the evaluated immune cells). A,** Hallmark enrichment analysis. **B,** C2 canonical pathway. *adjP, adjusted p-value; GSEA, gene set enrichment analysis; NES, normalized enrichment score.*


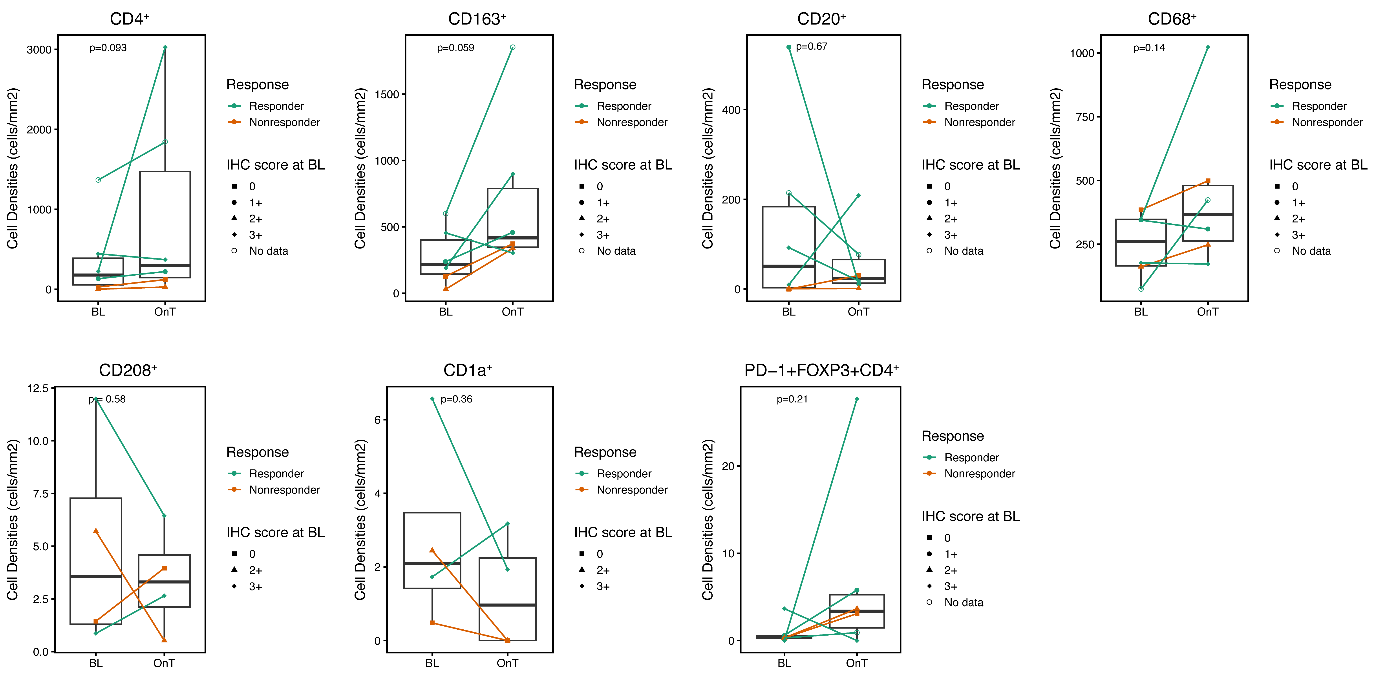


**Supplementary Figure S6. Modulation of tumor-infiltrating immune cell densities by T-Dxd.** *BL, baseline; IHC, immunohistochemistry; OnT, on treatment.*

**
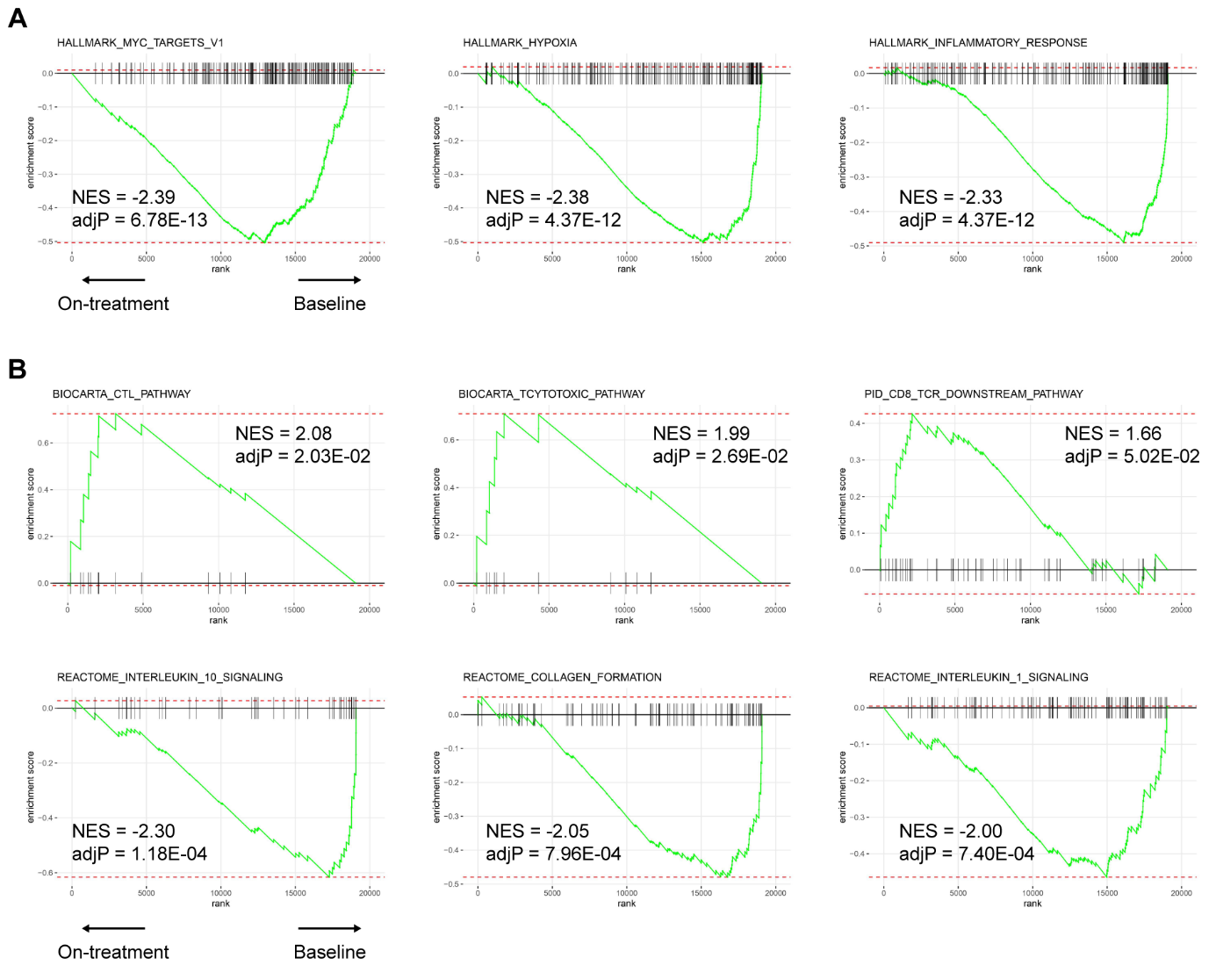
**

**Supplementary Figure S7. GSEA. A,** Hallmark enrichment analysis. **B,** C2 canonical pathway between baseline and on-treatment samples. *adjP, adjusted p-value; GSEA, gene set enrichment analysis; NES, normalized enrichment score.*
